# Supplementary material for: The Impact of Postoperative Complications on Survival after Simultaneous Resection of Colorectal Cancer and Liver Metastases
Source: Healthcare (Basel). 2022 Aug 19;10(8):1573. doi: 10.3390/healthcare10081573 (PMC9408276; doi:10.3390/healthcare10081573)
Supplement: Supplementary file 1 [file healthcare-10-01573-s001.zip › healthcare-1853222-supplementary.pdf]

Supplementary Table S1. Demographics, clinical, pathological and postoperative complications of the patients

Variable	Number of patients (%)
**Age**	
≤ 65 y-o	151 (62.1%)
> 65 y-o	92 (37.9%)
**Gender**	
Male	140 (57.6%)
Female	103 (42.4%)
**Primary tumor location**	
Right Colon	48 (19.7%)
Left colon	105 (43.2%)
Rectum	90 (37.1%)
**T stage**	
T1	1 (0.4%)
T2	6 (2.4%)
T3	216 (88.8%)
T4	17 (7.0%)
NA	3 (1.2%)
**N stage**	
N0	77 (31.7%)
N1	78 (32.1%)
N2	83 (34.2%)
NA	5 (2%)
**Distributions of SCLMs**	
Unilobar	176 (72.5%)
Bilobar	67 (27.5%)
**Number of SCLMs**	
1	137 (56.3%)
2-3	73 (30.1%)
≥ 4	33 (13.6%)
**Size of SCLMs**	
< 3 cm	126 (51.9%)
≥ 3 cm	117 (48.1%)
**TBS score**	
≤ 3.2	120 (49.3%)
> 3.2	123 (50.7%)
**Type of hepatectomy**	
Anatomic	34 (14%)
Non-anatomic	209 (86%)
**Extension of hepatectomy**	
Minor	211 (86.8%)
Major	32 (13.2%)
**Postoperative complications (Clavien-Dindo)**	
No complications	
I	121 (49.7%)

II	20 (8.3%)
III	48 (19.7%)
IV	35 (14.5%)
V	7 (2.9%)
	12 (4.9%)
**Septic complications**	
No	157 (64.6%)
Yes	86 (35.4%)
**Hepatic complications**	
No	184 (75.7)
Yes	59 (24.3%)
**Hepatic septic complications**	
No	203 (83.5%)
Yes	40 (16.5%)
**Comprehensive complication index** (median [IQR])	8.70 (0 - 26.20)
**Neoadjuvant treatment**	
No	210 (86.4%)
Yes	33 (13.6%)
**Adjuvant treatment**	
No	29 (11.9%)
Yes	214 (88.1%)
